# Seven years of studying the associations between political polarization and problematic information: a literature review

**DOI:** 10.3389/fsoc.2023.1174161

**Published:** 2023-05-11

**Authors:** Giada Marino, Laura Iannelli

**Affiliations:** Department of Economics and Business, University of Sassari, Sassari, Italy

**Keywords:** extremism, political polarization, radicalization, problematic information, disinformation, misinformation, fake news, literature review

## Abstract

This literature review examines the intersection between political polarization and problematic information, two phenomena prominent in recent events like the 2016 Trump election and the 2020 COVID-19 pandemic. We analyzed 68 studies out of over 7,000 records using quantitative and qualitative methods. Our review revealed a lack of research on the relationship between political polarization and problematic information and a shortage of theoretical consideration of these phenomena. Additionally, US samples and Twitter and Facebook were frequently analyzed. The review also found that surveys and experiments were commonly used, with polarization significantly predicting problematic information consumption and sharing.

## 1. Introduction

During the last seven years, the world has witnessed several unexpected political and social events that have left a lasting impact. From Great Britain's exit from the European Union, the election of Donald Trump as US President in 2016, the attack on Capitol Hill in 2021, to the COVID-19 pandemic, these events have shaken the world and tested the health of democracies (Allcott and Gentzkow, 2017; Tucker et al., 2017).

During these events, political polarization and problematic information have been identified as potential threats to the healthy functioning of democracy. Scholars and journalists have raised concerns about how these phenomena have worsened (Jack, 2017; Wilson et al., 2020), particularly with the increased social media news use.

The Trump era saw an asymmetric increase in extreme political attitudes among US elites and citizens (Faris et al., 2017) and the rise of the Alt-Right (Marwick and Lewis, 2017). However, political polarization is not new, particularly in the US. Scholars have long debated polarization as “culture wars” with alternative belief systems (Abramowitz and Saunders, 2008). Fiorina and his colleagues contended that political polarization had not increased during the 1980s and 1990s and that political differences between the general public and political elites were exaggerated (Fiorina et al., 2005). Conversely, the proponents of the “culture wars” theory found that Democratic and Republican voters expressed opposed positions on issues and ideologies, regardless of how extreme they were (Abramowitz and Saunders, 2008).

Even the COVID-19 pandemic has become a polarized arena of conflict in the United States (Allcott et al., 2020; Green et al., 2020) and Europe (Bobba and Hubé, 2021b), as governments and opposition took antithetical stances about the containment measures and the virus' origins, danger, diffusion, and therapy.

With the growing participation of citizens in the news cycle, particularly in the hybrid media system (Iannelli and Splendore, 2017), problematic information has increased in circulation, becoming another issue of concern for the health of contemporary democracies (Quandt, 2018). Problematic information is an umbrella term employed by Jack (2017) to indicate all those forms of information used to inflict harm on the audience, from completely false news and conspiracy theories to gaslighting information, propaganda, and post-truth.

An increasing asymmetric polarization and the wide circulation of problematic information seem less than ideal for well-functioning democratic processes (Tucker et al., 2018). Therefore, scholars have started to question whether and how these two phenomena interact and which consequences this interaction may have on democracies.

Although words like fake news, disinformation, polarization, and extremism are frequently cited in publications, they are sometimes used to describe social and political contexts rather than being studied directly. Additionally, while information issues are well-explored, political polarization is often used to describe unrelated phenomena such as selective exposure, distrust in news media and politics, and echo chambers.

The aim of the present study is to investigate the definitions, contexts, methods, results, and limitations that characterize the existing research on the relationship between political polarization and problematic information. The study reviewed scientific works published from 2016 to 2022, taking stock of what we know and the gaps that need to be addressed to understand this relationship.

The text discusses the motivation for this work by examining existing literature on polarization and problematic information, followed by detailing the workflow and analysis method in the method section. The study concludes with a description and discussion of findings, as well as recommendations for future research in the discussion and conclusions section.

## 2. Political polarization and problematic information, what we know and what instead is still missing in the existing literature

### 2.1. A matter of definitions

The association between political polarization and problematic information is not always easy to assess due to the complex nature of these two phenomena. Defining them systematically is difficult, as they encompass a variety of manifestations that are often challenging to place within scientific boundaries.

Regarding political polarization (Wojcieszak, 2015), there are at least four distinct forms: divergence, consistency, affective, and perceived (Lelkes, 2016). Polarization, or the state of being divided into two extreme poles, happens when opinions about political parties, ideologies, and specific issues become concentrated around those poles (Fiorina et al., 2005; Fiorina and Abrams, 2008). Divergence manifests as ideological extremism, radicalization, or hyper-partisanship, and it can be detected by the identification of bimodal or dispersed distributions in opinion scales (DiMaggio et al., 1996; Freeman and Dale, 2013; Pfister et al., 2013; Lee, 2016). Polarization as divergence can also be asymmetric, displaying as skewed distributions (see also Faris et al., 2017; Iannelli et al., 2022). Divergence can emerge in opinions about controversial and highly politicized issues that become an arena of conflict among political elites and their voters (Wojcieszak and Price, 2010; Lee, 2016; Wojcieszak et al., 2018; Iannelli et al., 2022). This form of polarization is labeled as “issue polarization” (Mason, 2015) or “issue-based extremity” (Wojcieszak and Rojas, 2011).

Consistency occurs when individuals take consistent positions across multiple lines of disagreement (Iannelli et al., 2021). Citizens' polarization thus happens when they align with opposite positions in areas of potential conflict, even if they don't take extreme stances (Abramowitz and Saunders, 2008; Baldassarri and Gelman, 2008). Conceiving political polarization as “consistency” means imagining citizens as divided into opposite factions with alternative and irreconcilable belief systems, with distinct manifestations such as “partisan sorting,” “issue partisanship,” and “issue alignment.” These forms of political polarization may increase political tension and hostility among citizens and potentially harm democracy, as noted by various scholars (Baldassarri and Gelman, 2008; Down and Wilson, 2010; Mason, 2015; Davis and Dunaway, 2016; Lelkes, 2016).

Affective polarization considers the dichotomy between emotions toward out-group and in-group members as a primary driver of political opinions (Iannelli et al., 2021). Affective polarization arises when individuals have strong negative emotions toward out-group members and positive emotions toward in-group members (Iyengar et al., 2012, 2019). This form of polarization is measured using a “thermometer of feeling,” which asks respondents to rate their feelings toward parties/leaders and their voters (Lelkes, 2016). Social polarization is a related phenomenon that occurs when inter-party hostility spills over into discriminatory behavior in interpersonal relationships, such as at work or in romantic relationships (Iyengar et al., 2012; Iyengar and Westwood, 2015). Both affective and social polarization have negative consequences for democracy and are measured through surveys that ask about respondents' attitudes toward individuals with opposing political views (Iyengar et al., 2012).

Lastly, perceived polarization refers to the extent to which citizens perceive the political system, the electorate, and/or the news media system as polarized (Levendusky and Malhotra, 2016; Yang et al., 2016; Vegetti et al., 2017).

The definition of mis/disinformative content is also complex due to its multifaceted nature, as it can take on multiple forms that are not entirely false but still harmful to the media ecosystem (Giglietto et al., 2019). These forms are not new but have been documented over time by scholars of information and politics (Lazer et al., 2018). With the advent of social media, the prevalence and level of dissemination of these informational disorders have changed, becoming a phenomenon of global interest. For this reason, recently, several attempts have systematized these types of content under a single term. The “Data and Society” team made one of the most widespread attempts to classify mis/disinformative content, and it has extensively studied the poisoned online news media environment and extreme users' media manipulation practices (Marwick and Lewis, 2017).

The study of false information has been refined in recent years by scholars who have sought to classify and understand the various types of problematic narratives circulating on social media. Jack (2017) distinguished between two types of false information: disinformation, the deliberate spread of false information, and misinformation, false information shared accidentally. Jack proposed a taxonomy of “problematic information” that includes conspiracy theories, rumors, propaganda, and completely made-up news. Similarly to Jack's work, Wardle and Derakhshan (2017) introduced the concept of “information disorder,” which covers a wide range of problematic news content, from satire to completely fabricated news. They also introduced the concept of “malinformation,” which refers to genuine information that is deliberately used to cause harm to the audience. The intersection of misinformation and malinformation leads to disinformation.

Giglietto et al. (2019) proposed a systemic approach to studying problematic information by observing problematic hybrid news cycles as an emergent system influenced by individual assessments and decisions.

To aid in the understanding and detection of online manipulation and disinformation operations, Donovan et al. (2020) created an online and open-access resource that provides a comprehensive list of definitions and related descriptions of practices and content for media manipulation on social media. This resource is useful for researchers and practitioners to map the landscape of online manipulation and disinformation.

### 2.2. The intersection of political polarization and problematic information in the existing literature

Despite growing attention on this topic, literature reviews on associations between political polarization and problematic information are few. Kubin and von Sikorski (2021) examined studies on social media's role in shaping political polarization and found that the consumption of pro-attitudinal social media exacerbates polarization. Kapantai et al. (2021) proposed a taxonomical review of studies on disinformation typologies and argued that new Big Data and Machine Learning content processing resulted in studies that are poor from a conceptual perspective. Jerit and Zhao (2020) observed that the existing literature on political misinformation has unevenly developed on psychological antecedents that lead to believing false news. Lastly, van Mulukom et al. (2022) considered studies on antecedents and consequences of COVID-19 conspiracy beliefs and found that they may lead to sympathy for violent radical actions, racism, and prejudices.

Tucker and colleagues' manuscript (2018) is one of the most cited literature reviews that focuses on the association between political polarization and problematic information. The third part of the paper section on polarization, misinformation, and the democratic process concentrates on the interaction between polarization and misinformation, which we are taking into account. Firstly, it deals with the issue of how partisanship and ideology affect belief in false and unsupported claims (motivated reasoning) by describing the case of misinformation about Obama and his presidency. Then it draws a relation between selective exposure and misinformation, i.e., polarization leads to selective exposure that, in turn, leads to false belief. Finally, it considers polarization as a sign of political distrust. By reading this literature review, we detected some gaps also in consideration of the high number of topics the paper deals with. The first and most evident regards polarization definitions that authors do not use in their multiple and different scientific meanings. Moreover, the study employs the political polarization label to indicate related issues that do not properly coincide with the phenomenon of polarization, such as selective exposure or distrust (e.g., “intensive polarized distrust”). This ambiguity in polarization definitions is not incorrect since the paper reports definitions of other studies that identify such phenomena as polarization. However, the authors did not introduce any warning about that.

Another widespread review of the existing literature in this field is that of Humprecht et al. (2020), in which political polarization is not the paper's primary focus. However, it is considered a key driver of the lack of resilience to believe in disinformation. The study aims to group eight Western countries based on their level of resilience to problematic information. Seven indicators from different data sources were used to conduct a cluster analysis. Negative levels of these indexes mean low resilience to disinformation. Conversely, high levels mean high resilience. The cluster analysis resulted in three groups. A cluster comprises countries resilient to disinformation (North Europe), while the other countries are non-resilient (South European countries and the US).

In their conclusion, the authors argued that, according to Benkler et al. (2018), media systems that are resilient to online disinformation are characterized, among other structural features, by a low degree of polarization. This low degree of polarization does not prevent citizens from coming across problematic information on social media but contributes to making them less inclined to believe and support it by further circulating it.

While this paper presents an original point of view based on the Hallin and Mancini (2011) geographical model, lacks a non-Western perspective. Moreover, it uses the phenomenon of polarization as one, among other structural features, leading to online disinformation diffusion. Not considering polarization as the primary focus of the study leads to an insufficient discussion dedicated to analyzing and thus understanding the relationship between political polarization and problematic information.

In conceiving the present study, we have thus taken into account the scarcity and gaps in reviews of the existing literature about the relationship between political polarization and problematic information. In order to surface, classify, and describe the main characteristics of this association, we formulated the following research questions:

RQ1. How are political polarization and problematic information defined?
RQ2. In what (geographical, temporal, media) contexts and with what methodological approaches are these two phenomena investigated?
RQ3. What are the relationships between political polarization and problematic information detected?
RQ4. What are the limitations of the existing studies?

## 3. Data and methods

### 3.1. References' identification protocol

The present study aims to examine studies that investigate the relationship between political polarization and problematic information. We conducted a systematic search on three leading academic databases, Google Scholar, Scopus, and Web of Science, limiting our search to a specific timeframe (from January 1, 2016, until July 31, 2022) due to the growing attention to these topics following the 2016 Brexit referendum, and the 2016 US Presidential elections.

#### 3.1.1. Query 1: search for keywords in the papers' abstract and body, if available

Based on literature about political polarization (Wojcieszak, 2015; Lelkes, 2016) and problematic information (Jack, 2017; Marwick and Lewis, 2017; Wardle and Derakhshan, 2017), we selected keywords for the search. Although we did not include “political” in our query (Table 1), studies in social and psychological sciences only consider political polarization's relationship with problematic information.

**Table 1:** Query string.

Extrem* OR hyperpartisan OR polariz* OR polaris* OR radicaliz* OR radicalis*	AND	Disinformation OR misinformation OR “fake news” OR misleading OR conspiracy OR rumors OR “problematic information” OR post-truth

We first attempted to search for studies about this issue in papers' abstracts and available bodies by using the Publish or Perish database query system. The result was 6,844 references. Considering the high number of retrieved references, we first filtered the dataset for the number of citations. We selected the ten most cited references for each year (*N* = 70). Nevertheless, at a preliminary examination, we observed numerous out-of-context papers and articles citing the query keywords just as buzzwords. A second attempt was made by filtering references for the mean of citation. We calculated the mean of citations in each year of publication and selected all references exceeding that yearly mean (*N* = 124). Despite this second attempt, the dataset's quality did not improve. We thus decided to exclude this dataset and try another query approach initially. This dataset will be later recovered.

#### 3.1.2. Query 2: search for keywords in papers' titles

Considering that the first ways of filtering did not comply with our expectations, we decided to narrow the search by repeating it with the exact query string, this time just questioning keywords in the title of the studies. In this way, we retrieved 283 references (Table 2). We will refer to this dataset as dataset 1.

**Table 2:** Results of the query in the title of papers.

**Total Retrieved Papers**		**283**
**Exclusion Criteria**	Duplicates	111
	Other languages then English	13
	Books or Book Chapters	19
	Thesis	17
	Newspapers or Blog Articles	3
	Other Publications (e.g., conference presentations, long abstract, etc.)	3
	Non-pertinent	14
**Total Clean Entries**		**103**

### 3.2. Data screening

In the first round of data screening, we worked exclusively on the dataset obtained by querying papers' titles and inspected the publication formats. We thus excluded duplicates, books, book chapters, thesis, newspapers, blog articles, or other types of publications such as conference presentations and posters. We also excluded non-pertinent records, that is, those not related to social sciences or in which the keywords have a different meaning from that intended by the authors (e.g., the word “extreme” to indicate “extreme climatic events”). We kept scientific journal papers, conference proceedings, scientific reports, and preprints in English. The results of this first screening are summarized in Table 2.

In order to have a more exhaustive dataset, we returned to the dataset of 6,844 records obtained by querying the abstracts. We categorized it entirely into eligible (*N* = 42) and non-eligible records (*N* = 6,814) by screening titles and abstracts and adopting the same criteria used in the screening of dataset 1. This subset retrieved from the first query's attempt will be named “dataset 2”.

Then we ran a second round of data screening in which the authors more closely inspected the papers. By examining the references' abstracts, we excluded publications that mention political polarization and problematic information only as buzzwords, without any attempt to research these two phenomena empirically and to understand the relationship between them. Additionally, we excluded reviews of existing literature from our analysis.

The references obtained after this screening process were 38 from dataset 1 and 30 from dataset 2. The final dataset included 68 records (Table 3 and Supplementary Datasheet 1).

**Table 3:** Summarized results of the data screening process.

Eligible references added from the dataset 1 (query in records' titles)	38
Eligible references added from the dataset 2 (query in abstract and body of article, if available)	30
**Final dataset**	**68**

**Figure 1:**
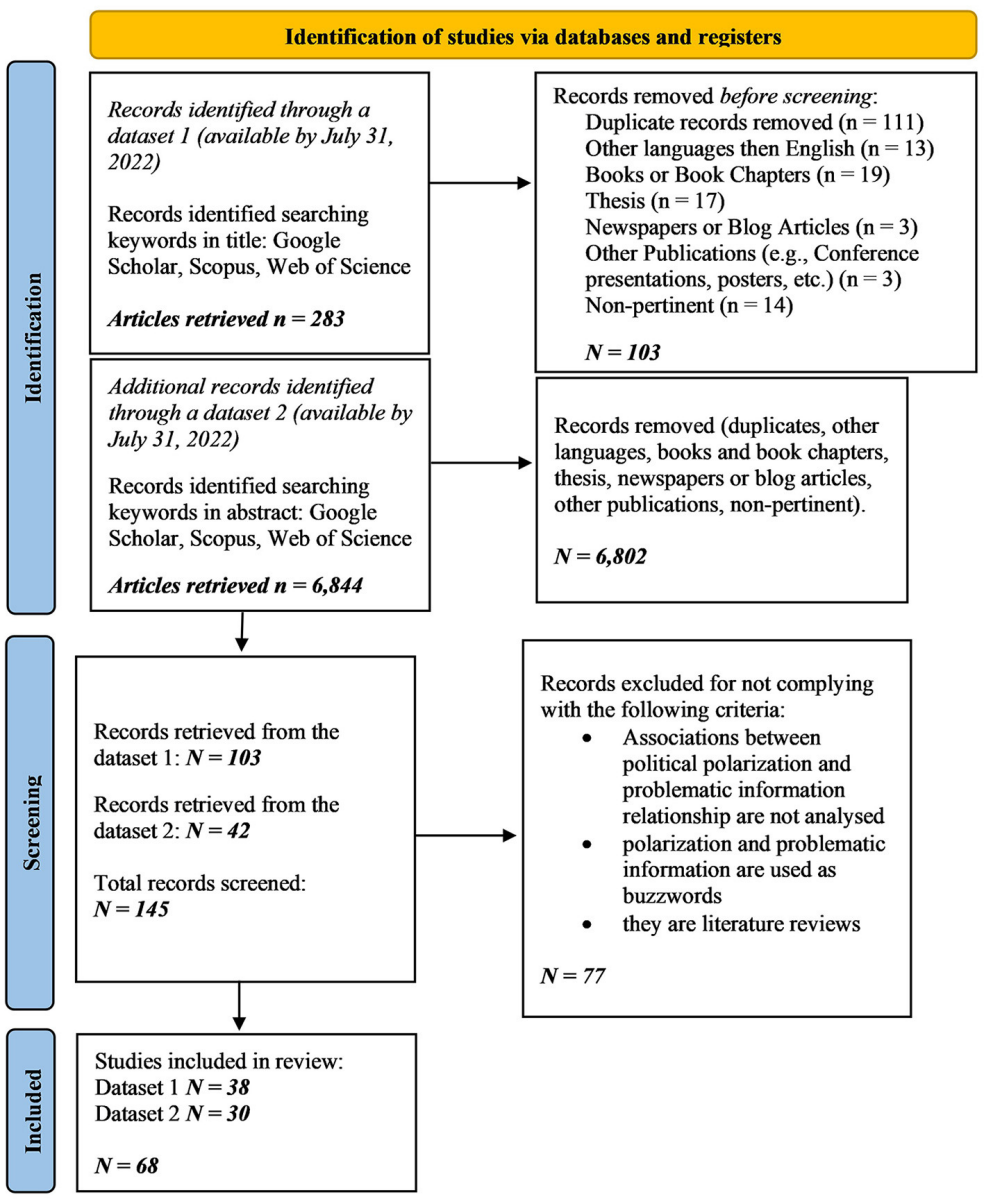
References query and screening based on PRISMA flowchart guidelines (Page et al., 2021).

The PRISMA flowchart in Figure 1 summarizes the whole process of identification and screening of the dataset.

### 3.3. Coding process

Following previous research works in the field (Humprecht et al., 2020; Kapantai et al., 2021; Kubin and von Sikorski, 2021), results were categorized using both quantitative and qualitative approaches. Content analysis was conducted without the support of machine learning.

To answer research questions, authors coded categories based on theoretical, contextual, and methodological aspects:

1. Whether the author(s) provided a definition of political polarization and problematic information, in what forms they have been analyzed, and if they have been studied at micro, meso, or macro level.
2. In which context(s) did the author(s) conduct the study (nation(s), timeframe, peacetime, or time of crisis, concerning newer or older media spaces), and how they studied the intersection between the two phenomena (theoretically or empirically, through quantitative, qualitative, or mixed methods, with what techniques, through a cross-sectional or a longitudinal design).
3. What (if any) relationships between political polarization and problematic information are detected.
4. Which limitations are made explicit by the author(s).

Papers were coded following a systematic codebook (see Table 4). To assess the coding strategy's reliability and content analysis results' consistency, the two authors coded a random sample of 7 papers of the dataset (around 10%). Then, the authors compared the two coded datasets. On this sample, we assessed the intercoder reliability. The agreement was 100% for most of the variables of the codebook, except for the following variables: political polarization definition (71%), problematic information definition (86%), methodological techniques (86%), and the direction of the association between political polarization and problematic information (86%). We discussed each discrepancy in this reliability sample until a consensus was reached. Moreover, after discussing discrepancies, the authors refined the coding protocol to define the instructions better. A single coder then coded the total pool of papers.

**Table 4:** Codebook.

**RQ1 - Theoretical aspects**	Do(es) the author(s) provide a definition of polarization?	• Yes • No
	Political polarization definition (provided by the author(s) or deduced from the measures)	• Divergence (asymmetric, extremism, hyperpartisanship, issue-based polarization, radicalization, etc.), • consistency, • affective (social polarization), • perceived, • other (which).
	Do(es) the author(s) provide a definition of problematic information?	• Yes • No
	Problematic information definition (provided by the author(s) or deduced from the measures)	• Conspiracy theories, • disinformation, • fake news, • misinformation, • rumors, • other (which).
	Micro, meso or macro phenomena	• Macro, i.e., phenomena detected at a societal-level. • Meso, i.e., phenomena detected at a group-level. • Micro, i.e., phenomena detected at individual-level.
**RQ2 - Methodological aspects**	Theoretical or empirical	• Theoretical studies. • Empirical studies.
	Methods and techniques	• Qualitative methods. • Quantitative methods. • Mixed methods.
		Primary technique: • content analysis, • experimental design, • focus groups, • qualitative interview, • (social media) big data analysis, • social network analysis, • survey, • other (which). Secondary technique, if any.
	Cross-sectional or longitudinal design	• Cross-sectional studies. • Longitudinal studies (replicated over months/years).
	Year of data collection	• Before 2016, • 2016, • 2017, • 2018, • 2019, • 2020, • 2021, • 2022.
	Elements of context	• Time of crisis. • Peace time.
		Eventually, which crisis? • Calamities, • climate crisis, • elections, • pandemic, • other (which).
	Media context	• Specific media are mentioned as the object of the analysis. • Specific media are not mentioned as the object of the analysis.
		Eventually, which media?
	Nations	Are one or more countries mentioned as the object of the analysis? • Yes • No
		Is this a comparative study? • Yes • No
		Eventually, which country/ies are mentioned?
**RQ3 - Findings**	Is an association/relationship between political polarization and problematic information detected?	• Yes • No
		Eventually, which association?
**RQ4 - Limitations**	Are limitations explicitly mentioned?	• Yes • No
		Eventually, which limitations?

### 3.4. Quantitative analytic strategy

To answer RQ1, we recorded whether the authors provided a definition of polarization or whether it was deducible from the measures used. We then classified it through preset labels grounded on the existing literature. Political polarization was classified as divergence, consistency, affective, perceived, and other forms. Problematic information's definition was classified through six labels: conspiracy theories, disinformation, misinformation, fake news, rumors, and other forms.

We then categorized the information concerning methodological aspects (RQ2). Firstly, we assessed whether or not a study was theoretical or empirical. Other information regarding the country of samples, the study's timeframe, the media analyzed, methodological approaches, design, and techniques were reported as mentioned by the authors in their studies.

### 3.5. Qualitative analytic strategy

In addition to the quantitative classification, we conducted a qualitative analysis of findings and limitations to answer R.Q.s 3 and 4. When available, we copy-pasted or summarized in sentences the findings and limitations of each paper. Regarding findings, we detected if a relation between political polarization and problematic information was found and in which direction. We did the same with limitations excerpts, detecting eventual trends and relations with studies nature, if any.

With these summaries, we also explored whether there were overlapping trends related to the intersections between political polarization and problematic information.

## 4. Findings

Despite the high number of records retrieved from two distinct datasets (see the methodological section), the number of eligible references was low (*N* = 68). This extensive improper use of keywords related to polarization and problematic information as research topics encouraged us about the necessity of a different approach, more focused on the studies investigating the intersections of the two phenomena. Using the two phenomena as buzzwords do not ease a better comprehension of the relationship between them and its implications for individuals and society.

In the next paragraphs, we will describe, quantitatively and qualitatively, the subset of studies regarding the relationship between political polarization and problematic information. In particular, we investigated definitions, contextual and methodological aspects, outcomes, and limitations.

### 4.1. Theoretical aspects

To answer RQ1, we explored how political polarization and problematic information are discussed and measured across all studies.

#### 4.1.1. Political polarization definition

Regarding polarization, the analyzed studies all include a specific definition or measure, enabling the classification of the studied phenomena. Table 5 demonstrates that most studies focus on political polarization as divergence, examining phenomena such as extremism, radicalization, and hyper-partisanship. This emphasis can be attributed to the prevalence of research in the United States (as noted below), where scholars have addressed far-right movements and asymmetrical divergence following Donald Trump's unexpected election (e.g., Faris et al., 2017).

**Table 5:** Number of papers by political polarization definition.

**Political polarization defined as…**	
Divergence (hyper partisanship, extremism, radicalization etc.)	46
Consistency	5
Affective polarization	4
Other (partisanship, echo chambers, selective exposure, controversies)	13
**Total**	**68**

Ten of the 47 papers on polarization as divergence address issue polarization. These papers measure polarization on controversial issues such as immigration and climate change using scales, indexes, or separate measurements. For example, Hameleers and van der Meer (2020) investigate the fact-checkers role in combating polarizing and misleading news through two experiments measuring polarization in attitudes toward immigration and the anthropic origin of global warming.

Three papers examine the intersection of problematic information and extremely intolerant attitudes toward Muslim communities. For example, Obaidi et al. (2022) find that endorsing the “Great Replacement” conspiracy theory is associated with radical anti-Muslim attitudes.

Five papers (Allcott and Gentzkow, 2017; Bowyer and Kahne, 2019; Pierri et al., 2020; Sutton and Douglas, 2020; Salvi et al., 2021) consider political polarization as consistency. They measure polarization as the alignment of extreme positions. For instance, Salvi et al. (2021) use socio-cognitive polarization to indicate citizens' consistency in extreme attitudes, like intolerance for ambiguity and xenophobia.

Four records examine affective polarization, often defined in these studies as opposite clusters of users disliking their political opponents, in line with previous research (Iyengar et al., 2012, 2019). For instance, Osmundsen et al. (2021) employed a positive and negative emotion scale to assess affective polarization and transformed it into two feelings scales, one for each political affiliation, measuring in-group and out-group levels of hate. One study in this group of papers measured social polarization; Kaiser et al. (2022) gauged users' intention to unfollow and block those perceived as sharing misinformation on a range intersecting with an ideological scale.

None of the records of the dataset mention perceived polarization.

Thirteen papers refer to political polarization as a different phenomenon. The majority of this subgroup (*N* = 6) defines it as partisanship (Benegal and Scruggs, 2018; Clayton et al., 2019; Ecker and Ang, 2019; Nielsen et al., 2020; Bai, 2021; Stecula and Pickup, 2021), while five others characterize it as echo chambers. For example, Del Vicario et al. (2016) examine the development of conspiracy communities on Facebook and describe echo chambers as “groups of like-minded people where they polarize their opinion” (p.1). This explanation is consistent with leading authors in digital media studies, including Sunstein (2018) and Barberá (2020), who argue that social networking sites allow politically like-minded individuals to cluster and be exposed to ideas that reinforce their viewpoints, leading to extremism. Nevertheless, several scholars have challenged this view and provided evidence that there is not necessarily a significant correlation between echo chambers and political polarization on social media (Dubois and Blank, 2018; Bruns, 2019; Iannelli et al., 2021).

While we were able to classify the definitions and measures of political polarization used in all papers based on seminal studies, it is worth noting that 27 studies lack a proper definition and rely heavily on detailed measure descriptions. This trend may be due to the prevalence of quantitative studies based on experiments and surveys, which we will explore in the following paragraphs.

Moreover, most of the theoretical foundations of these studies are based on the recent scientific literature on polarization, while not explicitly referring to the historical development of this phenomenon and its relationship with news media. Some studies, however, address this temporal issue (Clayton et al., 2019; Enders and Smallpage, 2019; Valenzuela et al., 2019; Rottweiler and Gill, 2020; Enders and Uscinski, 2021). Enders and Uscinski (2021), for example, introduce a theoretical framework on the relationship between political extremism and unsupported beliefs, also drawing on pre-2016 studies such as Lipset and Raab's (1978) work.

#### 4.1.2. Problematic information definition

Concerning problematic information definition, twenty-seven papers provide a theoretical definition of the phenomenon. We deduced the definition in the remaining forty-one from the measures and stimuli description provided.

Regarding problematic information sense in the studies under analysis, we found multiple definitions among those reported by Jack in her scientific report (2017) (see Table 6).

**Table 6:** Number of papers by problematic information definition.

**Problematic information defined as…**	
Conspiracy Theories/Beliefs	17
Misinformation	17
Fake News	16
Disinformation	7
Rumors	2
Others (false information, untrustworthy, anti-scientific, malicious, counter media content etc.)	9
**Total**	**68**

Among the records, seventeen mentioned conspiracy theories or beliefs. These were categorized as generic concepts under the term “problematic information” or specific popular conspiracy narratives, such as QAnon or the Great Replacement. For instance, Enders et al. (2022) conducted a longitudinal study spanning six years to investigate the primary ideological drivers behind support for the QAnon conspiracy theory. Unlike other studies linking asymmetric polarization to problematic information support, they found that both ideological extremes are associated with belief in this conspiracy theory.

Among studies focused on conspiracy theories, four, primarily in psychology, use the term “conspiracy beliefs” to describe suspicions of a group involved in secretive and malevolent goals (Krouwel et al., 2017, p. 438). Misinformation (*N* = 17) and fake news (*N* = 16) are other common labels. While the meaning of misinformation and disinformation generally overlaps, Rossini et al. (2021b) provide a specific definition of misinformation used by other authors (e.g., Fallis, 2015; Jack, 2017; Giglietto et al., 2019). They define misinformation as initially valid information later recognized as false or misleading and used this label in their study on dysfunctional information sharing, asking participants if they ever shared news they later discovered to be false (Rossini et al., 2021b).

In the papers that were analyzed, there were different ways in which the term “fake news” was operationalized. For instance, Guess et al. (2019) employed a blacklist obtained from BuzzFeed News to define problematic domains, whereas Borella and Rossinelli (2017) used a popular Swedish false news story about the prevalence of immigrants' sexual abuse in school as a stimulus. However, theoretical definitions of “fake news” were found to be scarce.

A few records mention problematic information as disinformation, rumors, or other labels such as false information, untrustworthy, anti-scientific, malicious, or counter-media content.

In line with the theoretical shortage, few studies introduce the topic of problematic information from a historical perspective (Krouwel et al., 2017; Enders and Smallpage, 2019; Rottweiler and Gill, 2020; Rousis et al., 2020; Enders and Uscinski, 2021). For example, Rottweiler and Gill (2020) draw from the classical scientific literature on conspiracy theories by Hofstadter (1964) and Goertzel (1994).

#### 4.1.3. Micro, meso, and macro level of the analysis

We then investigated whether and how the studies under analysis have defined or measured polarization and problematic information phenomena at different observational levels: that of the public opinion, which we defined “Macro” that of the investigated groups (“Meso” level), or as that of the single individuals (“Micro” level).

The analysis evidenced that most studies (*N* = 47) use phenomena definitions and measures at a micro level. In other words, they investigate relations between individual variables regarding attitudes and behaviors, such as citizens positioning themselves on an ideological scale or that have individually shared or consumed problematic news (see, for example, Allcott and Gentzkow, 2017; Chadwick and Vaccari, 2019).

Four studies employ political polarization observations as a meso-level phenomenon (Levendusky and Malhotra, 2016; Borella and Rossinelli, 2017; Cook et al., 2017; Riley, 2022; Schulze et al., 2022). These studies calculate group polarization measures ex-post by aggregating individual surveys or measuring the phenomenon in specific and circumscribed online communities.

Seventeen studies employ instead political polarization measurements or definitions at a macro level using variables and concepts concerning networks' properties and societal attitudes. It is the case, for instance, of theoretical papers that apply theoretical models to societal processes (see, for example, Kruglanski et al., 2022) or papers that defined polarization as echo chambers, measuring the properties of large groups of social media users (e.g., Del Vicario et al., 2016).

### 4.2. Methodological aspects

#### 4.2.1. Theoretical or empirical studies

Nine studies developed a theoretical model regarding on the intersection between polarization and problematic information, while a large majority employed empirical studies (*N* = 59).

When observing the relationship between political polarization and problematic information, theoretical studies in the dataset propose new analytical models that try to understand real-world phenomena or build on arguments, frames, and theories from preexisting field studies. For instance, a highly cited study by Spohr (2017) considers relevant factors that may drive ideological polarization by discussing other studies about two crucial cases, the 2016 Brexit referendum and the 2016 US presidential election. Another example of a theoretical model applied to problematic information and polarization is that of Sikder et al. (2020). Authors apply the framework of a social learning model to examine how it may change the way a networked society processes information, even problematic. This model can capture the effect of large-scale confirmation bias on social media in polarized countries.

### 4.3. Empirical studies

#### 4.3.1. Methods

As mentioned, empirical studies comprise most of the dataset. To assess the RQ2, we explored their main characteristics from contextual (country of the sample, period, and media involved, if any) and methodological perspectives.

Concerning the methodological approaches, in Table 7, we can observe that most of the studies employed purely quantitative methods (*N* = 54). While the remaining are almost equally distributed between qualitative and mixed methods approaches.

**Table 7:** Number of empirical studies per method.

Quantitative	54
Qualitative	3
Mixed	2
**Total**	**59**

#### 4.3.2. Techniques

Studies employing a survey as an inquiry method are about one-third of the total empirical records (Table 8). Studies employing experiments (*N* = 17), mostly integrated with survey data, follow.

**Table 8:** Number of empirical studies per technique.

Survey	19
Experiment	17
Qualitative and quantitative content analysis	11
(Social media) big data analysis	4
Social network analysis	4
Mathematical simulation	3
Focus group and media diaries	1
**Total**	**59**

Surveys are often conducted on representative samples of the general population recruited through public or private survey agencies. However, Bansal and Weinschenk's (2020) study diverged from this approach by using Amazon Mechanical Turk to recruit survey respondents. The study investigated how friend influence affects network polarization on social media by presenting participants with eight fake scenarios. Meanwhile, two other studies employing surveys, Hopp et al. (2020) and Osmundsen et al. (2021), focused solely on social media users using traditional recruiting methods based on social media user panels. Osmundsen et al. commissioned YouGov to recruit US Twitter users to investigate the hypothesis that attacking partisan opponents leads to problematic information sharing.

Among those studies using an experimental design (*N* = 17), several are secondary analyses based on findings of previous experiments. Bai (2021), for example, reviews five experiments conducted in the US to assess whether exposure to problematic information corrective actions can affect political attitudes revealing that the effect of misinformation exposure is almost resistant to fact-checking intervention.

In 11 content analysis studies, authors assess the relationship between political polarization and problematic information on social media content (e.g., news, comments) (Evangelista and Bruno, 2019; Hjorth and Adler-Nissen, 2019). Seven studies used automated content classification methods (e.g., topic modeling, machine learning, sentiment analysis) and claimed to be quantitative (Potthast et al., 2017; Del Vicario et al., 2019; Hjorth and Adler-Nissen, 2019; Mourão and Robertson, 2019; Baptista and Gradim, 2020; Rousis et al., 2020; Schulze et al., 2022), while four used qualitative approaches. Two of the four studies did not use machine support for analysis (Evangelista and Bruno, 2019; Hashemi, 2021), and the others used qualitative analysis as part of a mixed-method approach (Riley, 2022) or for machine learning algorithm training support (Recuero et al., 2020a).

We can find studies based on quantitative analysis of social media data. Four focus on analyzing social media content metrics of attention (*N* = 4) (Bessi et al., 2016; Ribeiro et al., 2017; Asadi, 2021; Phadke et al., 2022), while the other four employ social network analysis (Pierri et al., 2020; Recuero et al., 2020b; Restrepo et al., 2022; Weber et al., 2022). Three studies are based on mathematical simulations (Del Vicario et al., 2016; San Martín et al., 2020; Azzimonti and Fernandes, 2023). These studies employ mathematical models to reproduce real human interactions and behaviors.

One qualitative study by Bozdag and Koçer (2022) used focus groups and media diaries to analyze how political polarization affects perceptions of misinformation, sharing similar goals to those of quantitative studies. The lack of other qualitative research methods like semi-structured interviews and ethnography raises concerns about the absence of in-depth perspectives on individual behaviors and attitudes toward politics and news, as well as motivations behind polarization and problematic information consumption/circulation.

#### 4.3.3. Longitudinal or cross-sectional design

Most empirical studies are cross-sectional (*N* = 47), involving a single data collection. This prevalence of cross-sectional studies is likely due to the additional effort, in terms of time and funds, necessary to conduct a longitudinal study.

There are twelve longitudinal studies, mainly quantitative, based on different techniques, from survey to content analysis to social media big data analysis.

#### 4.3.4. Time of crisis

For “crises,” we intended potentially critical social and political events at a national or international level that disrupt normalcy, such as an election or the recent COVID-19 pandemic.

We observed that empirical studies conducted in times of crisis are 20. More specifically, studies focusing on general elections, particularly the US ones (Allcott and Gentzkow, 2017; Guess et al., 2019; Hopp et al., 2020; Hashemi, 2021; Riley, 2022), are equally distributed across years. We observe a general increase in studies focusing on times of crisis over the years. As expected, this increase may be due to the COVID-19 pandemic that has catalyzed the attention of researchers. From 2020, an increasing number of papers about the pandemic were published (Nielsen et al., 2020; Recuero et al., 2020b; Bai, 2021; Levinsson et al., 2021; Salvi et al., 2021; Restrepo et al., 2022).

#### 4.3.5. Media spaces

Regarding media spaces, fifteen studies don't focus on any media, while two analyze news media without specific internet references (Nielsen et al., 2020; Bozdag and Koçer, 2022). Forty-two studies analyze the internet or social media, eight of which generally mention “social media” without specifying platforms. These studies survey or experiment with social media users as respondents/participants (e.g., Valenzuela et al., 2019; Nagar and Gill, 2020).

Twitter (*N* = 10) and Facebook (*N* = 9) are currently the top-ranked social media platforms. Studies that analyze multiple platforms are rare: three have focused on two social media platforms, primarily Facebook and Twitter. Only a recent paper by Rossini et al. (2021b) examined the frequency of political talk and cross-cutting exposure on Facebook and WhatsApp, finding that the platforms' public vs. private nature affects the spread and mitigation of problematic information.

More in general, studies focusing on instant messaging services are still scarce. In our dataset, just three papers include WhatsApp in their analysis and one Telegram. Instead, other relevant social media platforms, such as Instagram and Tik Tok are completely missing.

#### 4.3.6. Country of sample

Thirteen studies are not country-specific. For example, they used Amazon Mechanical Turk or social media communities as recruiting systems.

Our analysis reveals an unsurprisingly disproportionate emphasis on samples from the United States (*N* = 22), given the research trends and recent events in the US involving political polarization and problematic information (Arnett, 2008; Wojcieszak, 2015).

Italy, Brazil, and Germany emerge as important countries of interest in the sample. The second-largest sample size, consisting of five studies (Bessi et al., 2016; Del Vicario et al., 2016; Zollo, 2019; Pierri et al., 2020; Salvi et al., 2021), comes from Italy, which is not surprising given the country's “pluralistic polarized” political and media systems (Hallin and Mancini, 2011) and its long-standing issues with social media, populist parties, and problematic information consumption (Rossi et al., 2021). Interestingly, half of the papers based on Italian samples also define polarization as echo chambers or selective exposure. Meanwhile, Brazil and Germany, which has the third-largest sample size split between them (four studies each), have both experienced a rise in far-right movements' popularity and related issues with political polarization and problematic information consumption. Brazil, in particular, has had a far-right government under Jair Bolsonaro that has aggressively promoted disinformation (Rossini et al., 2021a).

Five studies are comparative. Four compare two countries (Spohr, 2017; Zollo, 2019; Salvi et al., 2021; Obaidi et al., 2022), while one claimed to compare a 20 countries' scenario without mentioning any of those (Borella and Rossinelli, 2017).

Lastly, we would raise concerns about the lack of studies in this field focusing on non-Western countries or contexts where democracy is under threat, such as Russia, China, or African countries.

**Figure 2:**
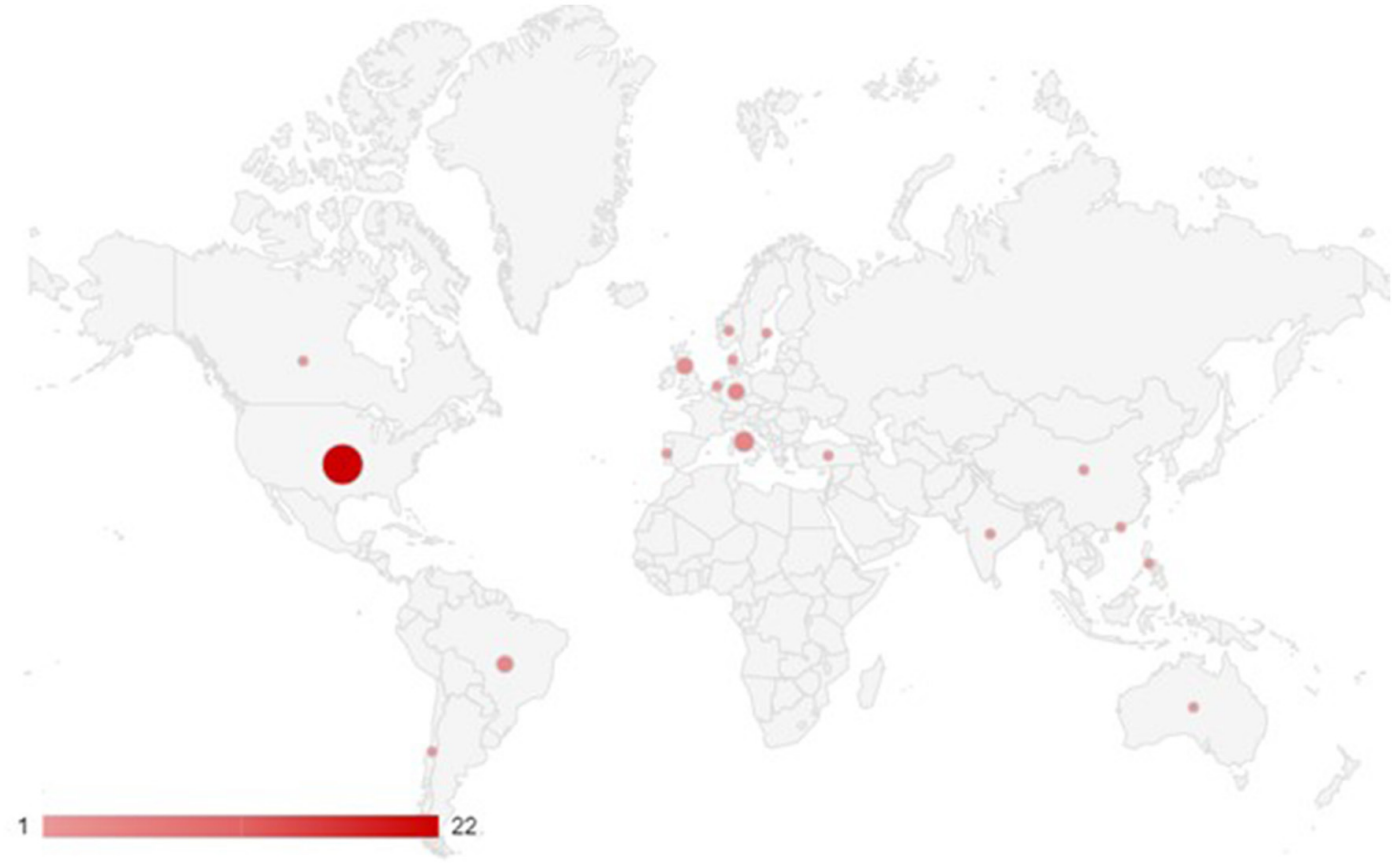
Nationality of the studies' samples.

Figure 2 shows the nationality of the studies' sample through a map. To build this map, each sample was attributed the value 1: the number of occurrences in the map is higher than the number of papers as five studies included more than one sample.

### 4.4. Main outcomes

Of the fifty-nine empirical studies that tried to find an association between political polarization and problematic information, thirty-eight have detected one.

Of the twenty-one not detecting any relationship, thirteen debate polarization as another phenomenon. Authors of eight studies have instead looked for this relationship while not joining any conclusions on this topic. For instance, Rossini et al. (2021b) analyzing predictors of incidental and purposeful misinformation sharing assesses that political extremism is not a significant antecedent of the dependent variables.

The outcomes of most of the studies thus suggest the presence of an association between political polarization and problematic information.

Among the studies suggesting this association, twenty-four are based on surveys and experiments conducted by scholars of diverse disciplines (political scientists, psychologists, media scholars, and economists). These studies tested their hypotheses of associations between polarization and problematic information through regression models. Then, we estimated the direction of the association by analyzing which of the two phenomena was employed as the antecedent of the other (used instead as the dependent variable) in the regressions' findings.

According to our analysis, only four of these experimental and survey studies suggest that problematic information is an antecedent of political polarization (Borella and Rossinelli, 2017; Cook et al., 2017; Rottweiler and Gill, 2020; Zimmermann and Kohring, 2020). Looking more in-depth at these studies, we can say that they suggest a polarizing effect of problematic information by investigating diverse aspects: the relations between false beliefs and voting for a far-right party; between conspiracy mentality and violent extremist intentions; between psychological mechanisms (such as the “backfire effect”) and extreme attitudes on current topics (immigration and climate change). Only one of these four studies employs a longitudinal research design (Zimmermann and Kohring, 2020), focusing on the 2017 German election campaign, when – according to the authors of this study – disinformation in online media presented a specific ideological orientation, given its negative framing regarding immigrants. In this contest, through a panel survey, Zimmermann and Kohring (2020) demonstrate that disinformation beliefs are fostered by distrust in the established political and information systems and that higher perceived believability of disinforming online news increases the likelihood of voicing this dissatisfaction by voting for the far-right party AfD.

Twenty studies explore the opposite relation by suggesting that political polarization is an antecedent of problematic information. Specifically, we observe that, in seventeen cases, ideological extremism and very strong partisanship positively correlate with believing in problematic information (Levinsson et al., 2021; e.g., Enders and Uscinski, 2021) or with sharing problematic information (e.g., Guess et al., 2019; Nagar and Gill, 2020). Of these studies, four provide evidence supporting a link between ideologically asymmetric polarization and problematic information. Specifically, these four studies suggest that individuals on the far-right are more prone to believing or sharing problematic information (Allcott and Gentzkow, 2017; Chadwick and Vaccari, 2019; Enders and Smallpage, 2019; Hameleers and Brosius, 2022). Lastly, three studies find a positive association between affective polarization and the probability of believing or spreading problematic information (Osmundsen et al., 2021; Hameleers and Brosius, 2022; Kaiser et al., 2022). Only two of these twenty studies employ a longitudinal research design (Guess et al., 2019; Enders et al., 2022). Using six public opinion polls conducted in the USA from 2018 to 2020, Enders et al. (2022) find that self-identified political extremism (both on the right and the left), antisocial psychological orientations, and non-normative attitudes are significant factors in QA non-support. In the context of the 2016 US Presidential Campaign, Guess et al. (2019) mixed self-reported data from a panel survey and behavioral data on the Facebook activity of survey respondents, showing that being very conservative increased the probability of sharing fake news on Facebook. According to the authors of this study, this result is consistent with previous political science studies favoring explanations based on stable ideological positions. However, given the overwhelming pro-Trump orientation in fake news via Facebook during that US Presidential Campaign, Guess, Nagler, and Tucker cannot rule out the possibility that the causal pattern they observed in this longitudinal study is due to “echo chambers” and the tendency of respondents to share articles they agreed with.

Eight studies use qualitative and quantitative content analysis as methodological approaches to demonstrate the relationship between political polarization and problematic information. These papers measure, for example, the problematic content prevalence in social media sharing practices of ideologically polarized groups of individuals (Hjorth and Adler-Nissen, 2019). They are also focused on information and news outlets' nature, such as the study of Recuero et al. (2020a), concluding that hyper-partisan news outlets share disinformation more often than others. Studies employing content analysis also detect associations between the two phenomena in communities related to specific fringe social media (Scott, 2022). For example, studies such as those of Schulze et al. (2022) and Riley (2022) analyze platform-based radicalized online communities, respectively, on Telegram and TheDonald.win, finding a proliferation of conspiracy narratives and misinformation.

The remaining six studies find an association between political polarization and problematic information, using social network analysis (*N* = 4), social media big data analysis (*N* = 2), and focus groups (*N* = 1) as analytical methodologies.

Studies based on social network analysis find that the circulation of problematic information is limited to certain clusters of extremist social media users. Pierri et al. (2020), for example, find that the dissemination of disinformation on Twitter was restricted to a small community that was strongly and explicitly tied to the conservative and far-right political sphere in Italy. Using a social media big data analysis approach, Phadke et al. (2022) find that individuals showing signs of radicalization do not restrict their interests to conspiracy theories, and Asadi (2021) demonstrates how the religious radicalism of Twitter users influences misinformation distribution. The sole qualitative study included in the dataset, conducted by Bozdag and Koçer (2022), show how the participants in their research displayed a high level of awareness regarding how the polarized media environment creates skepticism about the truthfulness of news.

### 4.5. Mentioned limitations

Studies explicitly reporting their limitations are 32. The presence of preprints, conference proceedings, and scientific reports in the dataset may partially explain this substantial lack of studies' limitations. Nevertheless, their number is far from constituting more than half of the records. Another reason, as aforementioned, may be found in the presence of theoretical papers and secondary analysis of experimental studies not reporting any explicit limitation.

Overall, studies analyzed often fail to report theoretical limitations. In quantitative studies utilizing experimental designs and surveys, limitations are often related to methodological issues such as sample size, recruitment methods, and representativeness. For instance, Valenzuela et al. (2019) noted limitations in their longitudinal approach and non-random assignment, while Benegal and Scruggs (2018) reported limitations due to the use of Amazon Mechanical Turk to recruit participants, which resulted in a non-representative sample.

Hameleers and van der Meer (2020) report design limitations, as they excluded participants with moderate attitudes and focused on only two highly polarized political issues. Rossini et al. (2021b) also report limitations in their study design, specifically the lack of objective measures for problematic information consumption or sharing, and self-reported data, especially those related to the evaluation of problematic news, may be subject to personal bias.

Studies based on content analysis report limitations in defining problematic information due to vagueness. Del Vicario et al. (2019) used the unofficiality of news sources to assess problematicness but acknowledged its limitations. Counterbalancing Rossini et al. (2021b) considerations, Riley (2022) pointed out that observational data alone doesn't provide a complete understanding of the association between political polarization and problematic information. This type of analysis does not fully capture motivations underlying specific behaviors or attitudes.

## 5. Discussion and conclusion

Recent research has given significant attention to polarization and problematic information, particularly regarding unexpected political and social events like Trump's election in 2016 and the Covid pandemic in 2020. However, there is an urgent need to understand better the associations between the two phenomena and their interference with democratic processes. While conducting our research, we found that few studies adequately addressed this issue, we noticed that studies adequately focused on this issue were still scarce, and existing literature reviews were not fully focused on the intersection of these phenomena. Thus, we aimed to fill the gap in literature review scarcity by assessing the state-of-the-art research in this field.

Our systematic literature review further confirmed our impressions of the studies' scarcity. Our data collection from three major scientific search engines (Google Scholar, Scopus, Web of Science) yielded 7,127 records. Of all these records, just sixty-eight were eligible for the analysis, which means they analyzed the associations between political polarization and problematic information.

An interesting finding was the lack of theoretical discussion regarding political polarization and problematic information as fundamental concepts in the studies analyzed. As also evidenced by Kubin and von Sikorski (2021) regarding polarization, we found that 13 studies describe polarization by referring to other phenomena, such as echo chambers or partisanship. Additionally, in most cases, we had to deduce polarization's definitions from the measures described in the papers.

The situation is no different for problematic information: it is operationalized in cases for survey questions or experiment stimuli, but theoretical considerations are often missing. This also applies to limitations, which mainly focus on sampling, neglecting methodological and theoretical issues.

The lack of theoretical frameworks in papers analyzing the relationship between political polarization and problematic information is a limitation for understanding and replicability of the analysis. We recommend that future research includes explicit and comparable definitions of these phenomena to build hypotheses or research questions, as suggested by other authors (Humprecht et al., 2020; Kubin and von Sikorski, 2021).

Regarding the literature's contextual elements, our quantitative analyses revealed a steep increase, from 2020, in research on the relationship between polarization and problematic information, due to the growing social divisions and infodemic observed across many societies during the COVID-19 pandemic (World Health Organization, 2020; Jungkunz, 2021).

The study also shows that US-centric research focuses on the intersection between political polarization and problematic information, as observed by previous scholars (Arnett, 2008; Wojcieszak, 2015). The overemphasis on the US also impacts the abundance of Twitter- and Facebook-based studies. This prevalence may be due to researchers' ease in retrieving data and their relevance in circulating problematic information. However, this homogeneity raises concerns about the generalizability of the findings to other regions and platforms.

While TikTok, a Non-Western growing platform in popularity and importance, is underlooked, a US-centric platform such as “The Donald.win” is studied, albeit in a single paper. Therefore, future research should consider geographical heterogeneity and regional social media to platforms' diversity to fully capture the complexity of the intersection between polarization and problematic information.

In general, studies based on those platforms that seem less appropriate for political participation are missing or are still scarce, for example, Instagram and Instant Messaging services. Instant Messaging services have millions of users worldwide. They could be key platforms in news consumption, sharing practices, and political participation (Iannelli and Marino, 2022) but, due to their semi-private nature, they are more difficult to investigate through observational methods.

Moreover, the review surfaced a critical lack of studies based on a more extensive media use for encountering and consuming news that should include “older” media as still relevant information access channels.

Future research should focus on other countries' societies and media spaces' role in influencing the relationship between political polarization and problematic information, as the available studies' results are still difficult to compare (Bos et al., 2016; Righetti et al., 2022). Moreover, we strongly encourage researchers to work with qualitative approaches for future studies. Indeed, despite its high time, funds, and human resources costs, qualitative research can provide a more in-depth view of the cultural processes and motivations that lead to assuming polarized attitudes and consuming/sharing problematic information.

Most studies have found an association between political polarization and problematic information. Among them, a larger body of research employed regressions on data collected through surveys and experiments, allowing us to estimate the direction of this association. According to our estimates, most of these studies based on regression models have hypothesized that polarization is an antecedent of problematic information. This predictive role of polarization is supported by extensive scientific literature predating 2016, as observed by Enders and Uscinski (2021) and Rottweiler and Gill (2020). Nevertheless, looking more in-depth at the surveys and the experiments we collected between 2016 and 2022, we can see that the hypotheses of directionality in the association between polarization and problematic information depend on what dimensions of these phenomena scholars empirically investigate.

The surveys and the experiments that have treated polarization as an independent variable (the majority) conceptualize and/or measure it in terms of ideological extremism, very strong partisanship, or feelings of inter-party hostility. These studies show that higher political extremism or affective polarization seems to increase individuals' probability of believing or sharing problematic information. However, since almost all these studies employed cross-sectional research designs, the issue of causality in this association remains unclear. The two longitudinal studies, conducted in the USA, observe a pattern where political extremism causes beliefs in QAnon conspiracy theory and fake news sharing on Facebook. This pattern tends to favor explanations of beliefs and behaviors based on stable, deeply held partisan or ideological predispositions, in line with political science classical and contemporary literature (e.g., Campbell et al., 1960; van Prooijen et al., 2015). Moreover, this pattern is supported by studies on confirmation bias, which have proven how truth judgments align with pre-existing political views (e.g., Bartels, 2002). According to the findings of these two longitudinal surveys, pre-existing radical political identities predispose individuals to believe in problematic information they agreed with and to share it.

The studies that have treated polarization as a dependent variable (the minority) conceptualize and/or measure it as a (proxy of) behavioral process (the probability of voting for extreme parties or of acting violently) or as issue polarization (opposite attitudes toward current issues). Following previous psychological studies on extremism (e.g., Bartlett and Miller, 2010) and adopting a cross-sectional research design, three of these studies suggest the effect of cognitive bias concerning problematic information on these forms of polarization. Specifically, the conspiracy mentality seems to foster violent extremist actions, and the exposure to fake news that contradicts established political beliefs – following a cognitive mechanism known as the “backfire effect” – seems to increase the polarization of attitudes toward immigration and climate change. The only longitudinal study using polarization as a dependent variable has been conducted during an electoral campaign in a multi-party system. In this case, in line with those media studies that frame disinformation as a factor aiming at destabilizing democratic processes (e.g., Bennett and Livingston, 2018), the scholars observe a pattern where disinformation beliefs are fostered by a breakdown of institutional trust and, in turn, foster the vote to extreme parties, through which this disaffection is expressed.

Given the scenario that emerged from our review, it is of fundamental importance that, in the future, scholars interested in the association between polarization and problematic information define what manifestations of these phenomena are going to be empirically investigated and conduct longitudinal studies to face the issue of causality of this association.

Moreover, our review points out that there is another topic related to problematic information which still needs to be further investigated. Studies analyzing social media platforms' attempts to mitigate the circulation of problematic information – for example, through banners matched with problematic content or their temporary ban – and those trying to understand the outcomes of individuals' verification and social correction behaviors on levels of political polarization are still a few. We recommend that future research dedicate more effort to exploring this association.

While this review provides valuable insights into the issue under analysis, it is not limitations-free. We focused solely on articles and preprints, excluding books and book chapters. Additionally, due to the numerosity of records, the dataset obtained from the query on the abstracts was classified just in eligible and non-eligible references, thus losing information on non-eligible cases, for example, on the prevalence of publication typology or contributions languages.

The task of studying how political polarization and problematic information impact each other is difficult, and it's likely to remain a challenge for future research. However, persisting in the research of these phenomena and how they worsen each other and affect democratic processes can lead to valuable insights into the most effective strategies to counteract this threat to democracy.

## Author contributions

GM and LI contributed to the conception of the study as well as the design of the codebook. GM worked on the dataset building, data screening, references coding, data analysis, and wrote the first draft of the manuscript. LI worked at the data screening and coded a sample of references and redefining the coding protocol. All the authors finalized all the sections of the manuscript and approved the submitted version.
